# Stretchable Piezoelectric Power Generators Based on ZnO Thin Films on Elastic Substrates

**DOI:** 10.3390/mi10100661

**Published:** 2019-09-30

**Authors:** Ioana Voiculescu, Fang Li, Glen Kowach, Kun-Lin Lee, Nicolas Mistou, Russell Kastberg

**Affiliations:** 1 Mechanical Engineering Department, City College of New York, New York, NY 10031, USArkastberg205@gmail.com (R.K.); 2 Mechanical Engineering Department, New York Institute of Technology, New York, NY 11568, USA; fli08@nyit.edu; 3 Chemistry and Biochemistry Department, City College of New York, New York, NY 10031, USA; gkowach@ccny.cuny.edu; 4 Mechanical Engineering Department, University of Montpellier, 34095 Montpellier, France; nicolas.mistou@etu.umontpellier.fr

**Keywords:** ZnO nanofilm, piezoelectric energy harvesting, stretchable power generator, wearable power generator

## Abstract

The paper describes a stretchable, microfabricated power generator that will be attached on the skin and will produce energy based on the movements of the human body. The device was fabricated on a polymeric, elastomeric, poly(dimethylsiloxane) (PDMS) sheet. It consists of a piezoelectric thin film of ZnO sandwiched between two stretchable gold electrodes. An innovative technique was used for the deposition of ZnO thin film on the gold electrode-coated polymeric substrate at low temperatures below 150 °C. This is the first attempt to use a uniform film of ZnO, for energy harvesting. The ZnO film had the thickness at the submicron scale and the surface at the centimeter scale. We demonstrated that under a strain of 8% the voltage output from this power generator was equal to 2 V, the power output was equal to 160 μW and the corresponding power density was 1.27 mW/cm^2^. This device has great potential for application in power sensors attached on the human body, such as temperature sensors or wearable electrocardiography systems.

## 1. Introduction

Flexible and stretchable electronics are attracting substantial attention because of their promising applications in many areas, such as wearable electronics, bendable displays, artificial skin, implantable biomedical devices, soft, biointegrated and bioinspired devices. The flexible electronics must not only bend but also stretch, compress, twist, and deform into complex, curvilinear shapes while maintaining levels of performance and reliability, similar to current, nonflexible electronic systems. The realization of fully integrated flexible or stretchable electronics demands to have an appropriate flexible or stretchable power supply device. Currently, there is high interest for energy harvesting devices that can replace the conventional batteries. There are many forms of energy sources in the surrounding environment, such as solar energy, mechanical energy, thermal energy, chemical and biological energy. Among these energy sources, mechanical energy is abundant and available and is specialized for human motion-related applications. Numerous researchers have focused on mechanical energy harvesting with piezoelectric materials as a source of energy for electronic circuits. The energy harvesting based on piezoelectric transducers transforms the mechanical energy absorbed by a transducer into electric voltage that is used for electric device actuation or stored in batteries for future use [1,2]. These harvesters are ideal to be used in micro-electromechanical systems (MEMS) applications. One of the most important MEMS structures for energy harvesting is the piezoelectric cantilever beam that generates energy because of the presence of ambient vibration [2,3,4,5,6,7].

Over the past ten years, stretchable electronics have been widely investigated by Rogers, who invented the stretchable electronics concept [8,9]. Moreover, stretchable devices are reported for monitoring heart function, stretchable temperature sensors, stretchable batteries, electrocardiogram (ECG) sensors and impedance spectroscopy sensors [9,10,11,12,13,14,15,16].

This research is focused on stretchable piezoelectric energy harvesting from movements of human body. The joints of the human body are in a continuous state of motion, providing excellent opportunities to harvest energy [17,18]. The device for harvesting energy from the human body has to be flexible or stretchable because the surface where the device will be attached is curvilinear. The key challenge for flexible energy harvesting devices is to choose the suitable materials with good flexibility and mechanical stability, as well as a design with a fully flexible structure. Single-crystal Si ribbons were demonstrated as functional for stretchable electronic transducers [19]. In this case a very thin film of single-crystal silicon was deposited on a prestrained polymeric substrate using conventional lithography process. When the polymeric substrate was released, the prestrain, the Si film had a wavelike geometry which can be stretched or flexed. 

The most common piezoelectric materials for flexible energy harvesting devices are lead zirconate titanate (PZT), zinc oxide (ZnO) and poly(vinylidene fluoride) (PVDF) [20]. Traditionally, lead zirconate titanate (PZT) has been the piezoelectric material commonly used for mechanical energy harvesting. Recently, several groups have demonstrated power generators for energy harvesting based on flexible PZT ribbons, which due to its reduced dimensions shows improved mechanical properties compared to the bulk thin-film [21,22]. This flexible nanogenerator fabricated from PZT ribbons can generate energy when its surface is flexed. The application is to harvest energy from heart movements [22]. The power density produced by this PZT nanogenerator is 0.12 μW/cm^2^, showing promise towards the implementation of wearable or implantable energy harvesters. However, the presence of lead in PZT piezoelectric ceramic raises potential issues in using these devices for long-term implantable applications.

Zinc oxide is a piezoelectric material that is non-toxic and biocompatible and is used in many applications of wearable or implantable devices. For harvesting energy applications, ZnO is grown as nanowire arrays on flexible polymer films [23]. Even one single ZnO nanowire was demonstrated as flexible nanogenerator. The piezoelectric nanowire was fixed at both ends to electrodes and laterally packaged on a flexible substrate. The bending of the substrate produced a tensile strain in the ZnO nanowire resulting in piezoelectric potential along the wire. The generated voltage and current were 20–50 mV and 400–750 pA [24]. The single ZnO nanowire was used for harvesting biomechanical energy into electricity from an oscillating human index finger movement [24,25]. This single ZnO wire was also implanted in a live rat and harvested energy generated by the heart beating [26].

Scale-up approaches were later developed to fabricate large areas covered with ZnO nanowires and enable simultaneous power collection from large amount of nanowires, by integrating top-down microfabrication with bottom up nanowire synthesis [27]. A high output with an open-circuit voltage of up to 2.03 V and a peak output power density of around 11 mW/cm^2^ was reported for a flexible high-output nanogenerator fabricated by a scalable sweeping-printing method [28]. In addition, recent advances in the development of high output nanogenerators use cone-shaped ZnO nanowires [29]. In this case low values of compressive stress of 0.11% produced an output voltage of 2 V. This is large enough to continuously power the operation of small electronic devices. Furthermore, an integrated nanogenerator based on densely-packed ZnO nanowires array on flexible substrate was developed with open-circuit voltage and short-circuit current ~58 V and 12 μA [30]. An extremely thin nanogenerator was fabricated from vertically aligned ZnO nanowires grown by solution chemistry on aluminum (Al) foil [31]. The super-flexible nanogenerator was attached on the skin of the eyelid, and its output voltage/current characterized the motion of the eye ball underneath.

Stretchable nanogenerators have been reported, for instance, a composite film-based piezoelectric energy harvester was fabricated from piezoelectric hemispheres [32]. The highly-ordered piezoelectric hollow hemisphere embedded composite was obtained by the deposition of ZnO or PZT thin films on a close-packed, monolayer, polystyrene (PS) bead template. This nanogenerator device produced the output voltage of ~4 V from convex bending. An ultra-stretchable piezoelectric energy harvester fabricated from very long silver nanowire percolation network (VAgNWPN) electrodes and ecoflex piezoelectric nanocomposite materials was also reported in the literature [33,34]. By 200% stretching stimulations, this ultra-stretchable device generated voltage of ~4 V and current of ~500 nA. 

**Figure 1:**
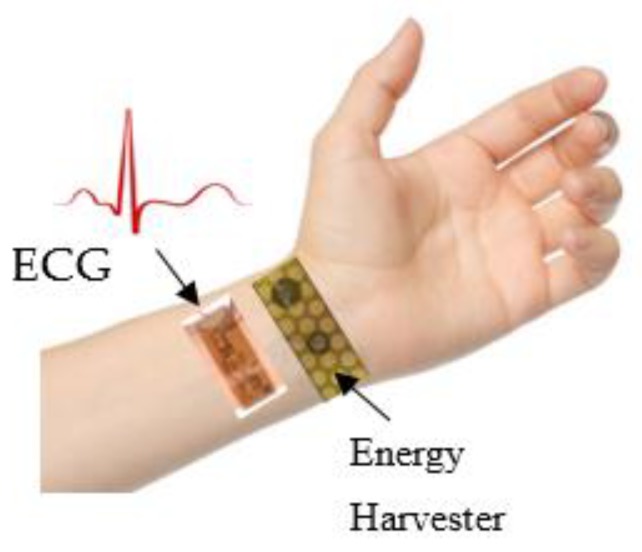
Illustration of a self-powered wearable electrocardiography (ECG) system. The ZnO energy harvester is intended to be placed on the skin of the arm.

The aim of this research is to develop a power generator that will be attached on the skin and will produce energy based on the movements of the human body. This power generator is intended to power sensors attached also on human body, such as temperature sensors or wearable electrocardiography (ECG) system. Figure 1 is an illustration of the position of the piezoelectric energy harvester on human hand. The movements of the arm articulation, see Figure 1, will stretch and bend the device that will produce power. The piezoelectric ZnO was chosen because is a biocompatible material, safe to be attached on the skin. According to literature the ECG system requires low power for its function of 13 μW/cm^2^ [35]. The stretchable power generator will produce an output voltage capable to power the ECG system.

The novelty of this research is to use a uniform film of ZnO deposited at low temperature on a large substrate area on the order of centimeter scale. The challenge of this research is to fabricate the ZnO film on a polymeric substrate without a significant reduction of piezoelectric performance. The usual deposition temperature for ZnO is around 400 °C even though higher deposition temperatures produce ZnO of higher crystalline quality. In the case of this research, the polymeric substrate will melt at these high temperatures. We used an innovative way to directly deposit ZnO on the polymer substrate below 150 °C. The stretchable Au electrodes for power collection were also directly fabricated on the polymer film. This is the first attempt to use a uniform film of ZnO for energy harvesting. This stretchable power generator based on ZnO film had the thickness at submicron scale and the surface at centimeter scale. We demonstrated that under a strain of 8% the voltage output from this power generator was equal to 2 V, the power output from this power generator was equal to 160 μW and the corresponding power density was 1.27 mW/cm^2^.

## 2. Materials and Methods

### 2.1. Fabrication of the Stretchable Power Generator

**Figure 2:**
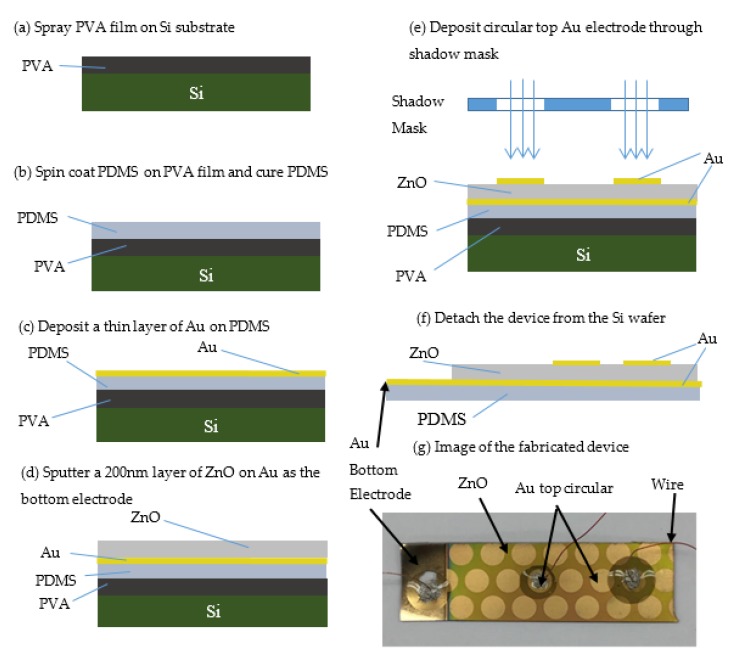
Illustration of the fabrication steps required for the power generator. (**a**) Spray polyvinyl alcohol (PVA) on a Si substrate. (**b**) Fabricate a layer of poly(dimethylsiloxane) (PDMS) on the PVA. (**c**) Deposit the bottom Au electrode for power harvesting on the PDMS. (**d**) Sputter ZnO on Au electrode. (**e**) Deposit circular Au electrodes on top of ZnO using a shadow mask. (**f**) Detach the power generator from the Si wafer by solving PVA film in water. (**g**) Photograph of the fabricated device.

The stretchable power generator, as shown in Figure 2a–g, consists of a ZnO piezoelectric thin film fabricated on stretchable polydimethylsiloxane (PDMS). One large gold electrode for energy collection was fabricated immediately underneath the ZnO film. Circular gold electrodes also for energy collection were fabricated on the top of thin ZnO film. To increase the active area of the device and improve the power output, we used a planar ZnO film. The thickness of the ZnO film was 200 nm.

A simple fabrication technique that started with a silicon wafer (1 0 0) (UniversityWafer, Boston, MA, USA) was developed. Regular microfabrication methods were used to clean the Si wafer. After the cleaning process, the Si wafer was dried on a hotplate at 110 °C for 2 min. Then, a thin layer of PDMS Sylgard 184 (Superior Essex, Atlanta, GA, USA) used as device substrate was fabricated on the Si wafer. In order to obtain simple detachment of PDMS layer from the Si wafer, a sacrificial layer of polyvinyl alcohol 98–99% hydrolyzed (PVA) (Alfa Aesar by ThermoFisher Scientific, Haverhill, MA, USA) was used between the Si wafer and the thin membrane of PDMS. The PVA was dissolved in water on a hotplate for 30 min at 80 °C, using a magnetic stirrer to facilitate the dissolution. A clear 5–7 w/v % PVA solution was sprayed on the Si wafer at room temperature (Figure 2a). The PDMS layer was deposited on the Si wafer covered with PVA using a spin coater (Laurell Technologies, North Wales, PA, USA) (Figure 2b). The ratio 10:1 (10 PDMS monomer and 1 curing agent) was used, and air bubbles were removed in a desiccator for 40 min. The viscous PDMS was spun on the Si wafer covered with PVA using 1000 rpm for 10 s and 1200 rpm for 1 min. PDMS was heated on the hotplate at 90 °C for 20 min. In this way, a PDMS layer with the thickness of approximately 100 µm was obtained. Four layers of PDMS were deposited to create a thicker membrane around 800 µm. On this PDMS membrane, a thin film of gold (Au) was fabricated as the bottom electrode for power collection (Figure 2c) using thermal evaporation method. The deposition of Au was performed at about 150 °C, and the PDMS material was able to withstand this temperature without warping. The bottom Au electrode covered the entire PDMS surface. The ZnO fabrication was performed in custom-made sputtering equipment developed at The City College of New York. A 3-inch target of pure Zn 99.995% (PURE TECH Inc., Centerville, MA, USA) was installed on the cathode. A power of 200 W (pulse DC sputtering, ENI RPG50 generator, ENI, Rochester, NY, USA) was used to reactively sputter Zn using only oxygen gas (O_2_) at a pressure of 15 mTorr. Argon was not employed during the sputtering process. The deposition was performed without external heating, but the sample reached a temperature of nearly 150 °C by the completion of the deposition. (Figure 2d). A deposition time of 25 min resulted in a ZnO layer with a thickness of approximately 200 nm. 

After the ZnO thin film was deposited, circular Au electrodes for power collection were deposited by using thermal evaporation (Figure 2e). The circular configuration of the top Au electrodes was achieved using a shadow mask, which had circular cuts of 4 mm diameter. The last step was the PVA dissolution in water at room temperature. The device was detached from the Si wafer, as illustrated in the Figure 2f. Figure 2f is the cross section of the fabricated device. The substrate was fabricated from PDMS that was covered with the bottom Au electrode for power collection. The ZnO film did not cover the entire bottom Au electrode. The area of the Au bottom electrode not covered with ZnO was accessible for attaching the wires to this bottom electrode, see Figure 2f,g. The top Au power collection electrodes were deposited at the end of the fabrication process. In this way all the wires were attached on the upper side of the device. For testing, commercial wires were connected to the top and bottom power collection electrodes with conductive glue. We used PDMS to cover the conductive glue to protect the connections. 

### 2.2. Characterization of the ZnO Thin Film

The crystal structure and morphology of the ZnO thin film sample were characterized with X-ray Diffraction (PANalytical X’Pert PRO, Panalytical, Almelo, The Netherlands) and Scanning Electron Microscopy (Zeiss SUPRA 55VP, Carl Zeiss, Jena, Germany), respectively. The thickness of the ZnO layer was measured by reflectometry (SCI FilmTek 3000SE, Film Tek^TM^, High Point, NC, USA).

### 2.3. Testing of the Electrode Resistance under Stretching

**Figure 3:**
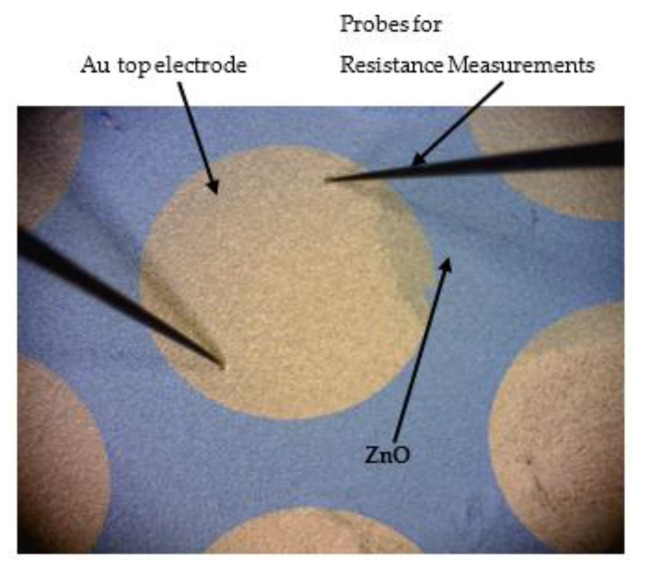
Gold power collection electrodes resistance was measured during stretching using a probe station.

The device is stretchable and formed by thin films of Au and ZnO integrated on PDMS polymer. In order to not damage the ZnO piezoelectric film, the maximum strain applied on the power generator was limited to 8%. We measured the variation of the resistance for the gold film, applying two repeated cycles of stretching with 1%, 2%, 3%, 4%, 5%, 6%, 7% and 8% as maximum strain. A probe station (Cascade Microtech GmbH, Dresden, Germany) was used for these resistance measurements. Special probes in form of needles were used to measure the resistance variation of the Au electrodes, when the power generator from Figure 2 was exposed to cyclic stretching. Two needle probes were placed on top of Au electrode, see Figure 3.

### 2.4. Testing the Power Generation of the Device

For testing the stretchable power generator device, a simple longitudinal gliding system was used to elongate/stretch the prototype with a stepper motor, M-23 NEMA 23 6.0A with a step of 1.8° (Schneider Electric Motion, Andover, MA, USA). The forward and backward movements of the stepper motor allowed the gliding system to produce a smooth linear translation. These linear movements of the gliding system produced stretching with controlled strain percentage and returned to the initial non-stretched position of the prototype accurately. This stepper motor was chosen because it was capable of high microstep resolution. The position and the speed of the motor were controlled by Uno R3 Arduino card and the software Arduino (Arduino^®^, Evrea, Italy). Using a worm screw that was rotated by the stepper motor, the linear position of the lower jaw of the gliding system was monitored. A LabVIEW program (National Instruments, Austin, TX, USA) was conceived to control the movements of the stepper motor. First, a digital oscilloscope (TBS1032B Tektronix, Tektronix, Beaverton, OR, USA) was used to measure output voltage from the power generator. The power generator was situated on the gliding system of the stepper motor and connected to an oscilloscope using the bottom and top collection electrodes. The output voltage measured with the oscilloscope was 2 V. The frequency of elongating/stretching the prototype of the stepper motor was 1 Hz and the elongation strain was 8%.

After the oscilloscope measurements proved that the power generator is producing a voltage, the output power was measured using a Keithly 2400 source meter (Keithley, Solon, OH, USA). Using the source meter controlled by the LabVIEW program, the stretchable device output voltage and current and calculated output power under different strain conditions were recorded. The device was connected straight to the source meter using the two wires connected to the upper and bottom electrodes. The Arduino software allowed the control of the gliding system movements and the LabVIEW program recorded the measurements of voltage and current from the Keithly source meter. In order to increase the accuracy of the measurement, the algorithm used was a loop with all devices. For each loop, LabVIEW gave an order to the motor to run a defined number of steps which corresponded to a certain elongation. For each step, the elongation was 0.0225 mm. 

To improve the accuracy of the measurement, a displacement sensor was fixed on the gliding system between the two jaws (moving and fixed). The sensor used was VL53L0X time-of-flight-ranging sensor (STMicroelectronics-Mouser Electronics, Geneva, Switzerland). This sensor has an embedded infrared, eye-safe laser for displacement measurements and works with an accuracy of 4%. This accuracy can be an inconvenience for long distance measurement, but for elongation up to 5 mm this error is acceptable. 

## 3. Results and Discussion

### 3.1. ZnO Thin Film Properties

**Figure 4:**
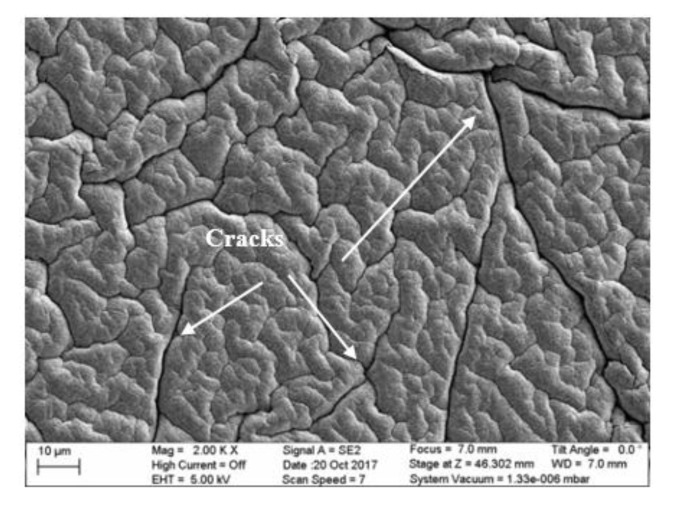
Scanning electron microscope (SEM) image of ZnO film.

A scanning electric microscope (SEM) image of the ZnO thin film is shown in Figure 4. The ZnO film had some cracks due to the stretchable and flexible nature of the PDMS substrate. However, the Au top electrode was deposited before the appearance of the cracks to ensure that the device did not have a short circuit due to contact between the top and bottom electrodes. Cracking was observed along the grain boundaries, but this does not diminish the piezoelectric properties since the film is oriented with the *c*-axis normal to the substrate.

### 3.2. Resistance of the Gold Electrodes under Stretching 

**Figure 5:**
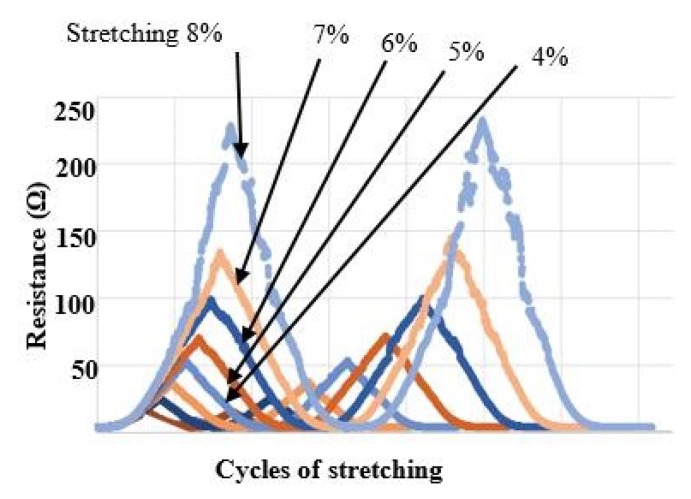
Variations of the Au resistance during stretching from 1% to 8%. Two stretching cycles are presented.

The variation of the resistance of the gold film, applying two repeated cycles of stretching with variations of strain from 1% to 8%, was measured. During stretching there was a maximum resistance variation of approximately 230 Ω corresponding to 8% strain, see Figure 5. Another observation is that the resistance values corresponding to different degree of stretching are similar for cycle 1 and cycle 2. Thus, the values of resistance of Au electrodes during stretching with different strain rates are changing, but the variations are repeatable and depend on the strain rate values. 

### 3.3. Power Generation Performance

**Figure 6:**
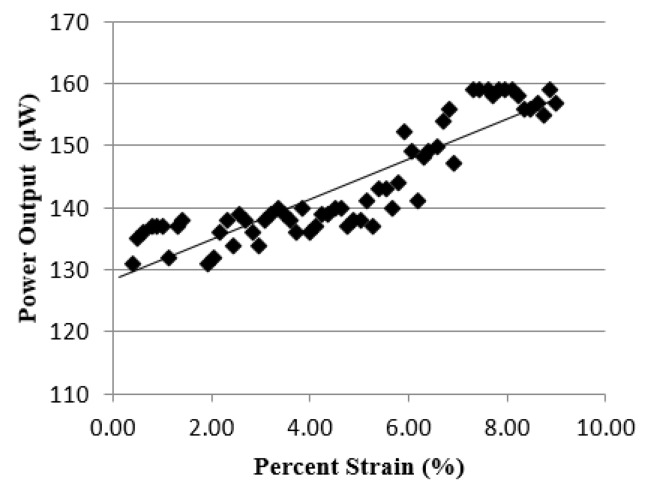
Power output versus power generator elongation. The initial experimental strain was set to 9%. We observed that starting from the strain of 8% the device output power was constant, with the value equal to 160 μW.

Initially, the device was elongated to a strain of 9% while the stretching frequency was set to 0.5 Hz. The power versus elongation increased in linear fashion up to the strain 8%, see Figure 6. Strains of greater than 8% led to a constant power output. Therefore, all samples were elongated with the maximum strain of 8%. For this strain value, the power output was 160 μW. 

**Figure 7:**
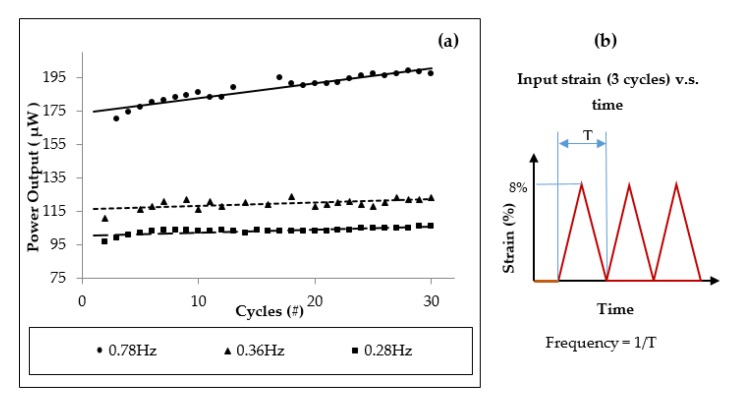
Power output versus elongation frequency (Hz). (**a**) The nanogenerator elongation was performed with three different frequencies; 0.78 Hz, 0.36 Hz and 0.28 Hz. As expected, the higher frequency will deliver the higher power. The elongation cycles (repetitions) were performed from 3 cycles to 30 cycles with different frequencies. In the case of higher frequency and higher number of cycles, the power increased. (**b**) Explanation of the elongation frequency. The frequency is considered equal to the number of device stretch/release per second.

The power generator was tested with different frequencies to see if the power output is influenced by the frequency of elongation. The device was elongated to the maximum strain of 8%. The frequencies for these tests were: 0.78 Hz, 0.36 Hz and 0.28 Hz, see Figure 7a. Figure 7b illustrates the elongation frequency for three cycles of stretch/release of the prototype. The frequency is considered equal to the number of device stretch/release per second.

Cycles (repetitions) from 3 cycles to 30 cycles were used with different frequencies of elongation. The corresponding power values at lower stretching cycles of 3 cycles were 100 μW, 115 μW and 175 μW. At the highest frequency of 0.78 Hz for stretch/release applied on the power generator and at highest cycles of 30 cycles, the power output reached 200 μW. These tests demonstrated that the higher power output was generated by the highest stretching frequency and using a higher number of cycles. Long-term reliability tests were not conducted; however, devices were remeasured over the course of a year for about 30 min for each test, and the results did not show degradation.

**Figure 8:**
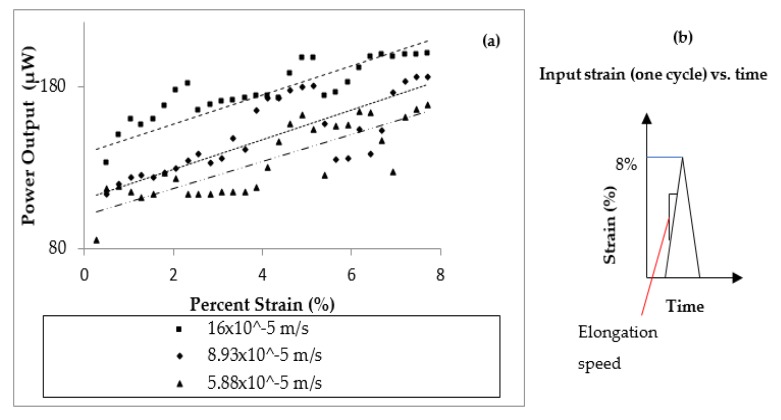
Power output versus elongation speed (m/s). (**a**) Three different elongation speeds were chosen. It was demonstrated that the highest elongation speed will provide the higher power output. (**b**) Explanation of the elongation speed. The device was elongated at the maximum strain of 8% with three different values of the speed.

The effect of the speed used for device stretching on the output power, see Figure 8, was also studied. The maximum strain for this experiment was 8%. The power generator device was elongated one time, so the number of cycles was 1, with three different speeds; 16 × 10^−5^ m/s, 8.93 × 10^−5^ m/s and 5.88 × 10^−5^ m/s. In this case the device was elongated only one time, and thus different frequencies of elongation or different elongation cycles are not relevant. As explained before, the gliding system used for testing was equipped with a displacement sensor, capable to record the speed, especially for this type of experiment. From Figure 8a, it was observed that the value of the stretching speed influenced the output power. For faster stretching speeds, the output power is higher, see Figure 8a. The maximum power output reached 200 μW. Figure 8b illustrates the concept of elongation speed.

These experiments indicate that the power output linearly increases with the tensile speed. This is the velocity used to stretch the device. The results from Figure 8 show that the performance characteristics of the ZnO thin film are consistent with fundamental piezoelectric theory [36]:

$$i=d_{33}EA\dot{\varepsilon }$$

where *i* is the output current; *d*_33_, *E* and *A* are the piezoelectric charge constant, Young’s modulus and cross-sectional area of ZnO film, respectively, and $\dot{\varepsilon }$ is the applied strain rate on the stretchable device which is proportional to the tensile speed. The current output is larger when the device is elongated at higher speeds.

## 4. Conclusions

Novel designs, device architecture and materials for piezoelectric energy-harvesting devices offer potential benefits but also challenges for device testing and characterization.

In this paper, a stretchable power generator, based on a novel ZnO film deposited at low temperature, is presented. Electrodes were fabricated using Au for power harvesting from the ZnO film, and PDMS was employed as an elastic substrate for the device. A power output from this power generator was found to be 160 μW for a strain of 8%, and the corresponding power density was 1.27 mW/cm^2^. The output power can be increased by increasing the frequency of elongation or the elongation speed. In this research the frequency of elongation from 0.28 Hz to 0.78 Hz was measured. 

The energy harvested by this device will be able to power wearable skin sensors. In the future reliability testing of the power generator including long-term stability and fatigue testing will be performed to study this device over time and with usage.
